# Using Deep Learning with Convolutional Neural Network Approach to Identify the Invasion Depth of Endometrial Cancer in Myometrium Using MR Images: A Pilot Study

**DOI:** 10.3390/ijerph17165993

**Published:** 2020-08-18

**Authors:** Hsiang-Chun Dong, Hsiang-Kai Dong, Mu-Hsien Yu, Yi-Hsin Lin, Cheng-Chang Chang

**Affiliations:** 1 Department of Obstetrics and Gynecology, Tri-Service General Hospital, National Defense Medical Center, Taipei 114, Taiwan; surgjimmy@yahoo.com.tw (H.-C.D.); hsienhui@ms15.hinet.net (M.-H.Y.); 2 Department of Public Administration & Taiwan Institute for Governance and Communication Research, National Chengchi University, Taipei 116, Taiwan; hkdong@nccu.edu.tw

**Keywords:** artificial intelligence, endometrial neoplasms, magnetic resonance imaging (MRI), neoplasm staging, neural networks (computer)

## Abstract

Myometrial invasion affects the prognosis of endometrial cancer. However, discrepancies exist between pre-operative magnetic resonance imaging staging and post-operative pathological staging. This study aims to validate the accuracy of artificial intelligence (AI) for detecting the depth of myometrial invasion using a deep learning technique on magnetic resonance images. We obtained 4896 contrast-enhanced T1-weighted images (T1w) and T2-weighted images (T2w) from 72 patients who were diagnosed with surgico-pathological stage I endometrial carcinoma. We used the images from 24 patients (33.3%) to train the AI. The images from the remaining 48 patients (66.7%) were used to evaluate the accuracy of the model. The AI then interpreted each of the cases and sorted them into stage IA or IB. Compared with the accuracy rate of radiologists’ diagnoses (77.8%), the accuracy rate of AI interpretation in contrast-enhanced T1w was higher (79.2%), whereas that in T2w was lower (70.8%). The diagnostic accuracy was not significantly different between radiologists and AI for both T1w and T2w. However, AI was more likely to provide incorrect interpretations in patients with coexisting benign leiomyomas or polypoid tumors. Currently, the ability of this AI technology to make an accurate diagnosis has limitations. However, in hospitals with limited resources, AI may be able to assist in reading magnetic resonance images. We believe that AI has the potential to assist radiologists or serve as a reasonable alternative for pre-operative evaluation of the myometrial invasion depth of stage I endometrial cancers.

## 1. Introduction

Endometrial cancer is one of the leading gynecologic malignancies in industrialized countries. The incidence of endometrial cancer has been increasing significantly worldwide in the past 10 years [1,2]. Myometrium, the middle layer of the uterine wall, serves as a barrier to prevent further expansion of endometrial cancer [3,4]. When the disease is diagnosed at an advanced stage, poor prognoses can be expected. One of the key parameters used to determine the stage is the depth of myometrial invasion. This is a prognostic factor used to categorize patients into high or low–intermediate risk categories, leading to different postoperative treatment approaches [5]. Therefore, accurate diagnoses followed by appropriate treatments in the early stages are the keys to good prognoses [6,7,8]. Currently, magnetic resonance imaging (MRI) is the primary tool used to evaluate the depth of myometrial invasion in endometrial cancer [9,10,11].

With the development of artificial intelligence (AI), radiologists have begun to use this technology to read medical images for various diseases [12,13,14]. AI comprises a collection of algorithms, mathematical functions, interrelated practical approaches, and overlapping areas of mathematics and statistics, which are well-suited for radiology because the pixel values of an MRI image are quantifiable [14,15]. Artificial neural network (ANN), for instance, is one technique used in the subdiscipline of classification systems. In ANN, the idea of deep learning (DL) has gained considerable attention. Various types of sub-algorithms concerning advances in fast processing, memory enhancement, and new model features and designs are continually being developed and upgraded [15]. The most common ANN used by DL is the convolutional neural network (CNN), which is the most suitable neural network for radiology when images are the primary units of analyses [15,16]. CNN is biologically inspired network mimicking the behavior of the brain cortex, which contains a complex structure of cells sensitive to small regions of the visual field. CNN not only comprises a series of layers which maps the image inputs to desired end points, but also learns higher-level imaging features [17].

According to the International Federation of Gynecology and Obstetrics (FIGO) classification system, endometrial cancer can be categorized as Stage I to IV, and Stage I can be further separated into IA and IB, which are distinguished based on the depth of myometrial invasion (less than vs. more than 50% myometrial invasion) [6]. However, the ability to determine the pre-operative MRI stages based, mainly, on personal expertise and experience which vary dramatically from person to person [18]. Additionally, various pathological factors—such as hematometra, interference due to a large coexisting leiomyoma or adenomyosis, or differences in the histological subtypes of the endometrial carcinoma—may lead to incorrect myometrial invasion diagnoses [10,19]. Discrepancies often exist between the pre-operative MRI staging and the post-operative pathological staging.

The previous literature about AI assistance in endometrial cancer diagnosis focuses on the performance of “post-operative” diagnosis (histopathological hematoxylin and eosin image) made by CNN-based classifier [20], while research examines the “pre-operative” MRI staging and performance of AI interpretation on endometrial cancer is rare. Our study is the pilot one to examine whether AI has the ability to assist physicians in making diagnoses of MRI before invasive surgery, i.e., pre-operative diagnosis. To achieve this goal, we compared the myometrial invasion diagnostic accuracy rate of the DL model with that of radiologists. Here, we used CNN to identify the myometrial invasion depth of endometrial cancer at an early stage and discussed the implications using AI as an auxiliary resource for making more comprehensive judgements.

## 2. Materials and Methods

### 2.1. Study Population

This is a retrospective study examining data from 72 endometrial cancer patients who received surgical treatment. Originally, there were 262 patients who had surgeries at the Tri-Service General Hospital in Taipei, Taiwan from January 2014 to September 2018. However, since we were interested in examining the ability of AI to validate myometrial invasion in early stage cancer, we excluded patients whose endometrial cancer was staged based merely on post-operative pathology without preoperative MRI scans and patients with stage II, III, and IV cancer. Eventually, 72 patients were qualified for this study (see Table 1). Among these, 53 were diagnosed with stage IA cancer, and 19 were diagnosed with stage IB based on permanent pathology. The average age of the patients was 59.7, with a minimum age of 39 and maximum age of 85. In terms of menopausal status, 63 (87.5%) postmenopausal. In terms of histology grade, 27, 32, and 13 belonged to grades 1, 2, 3 (5 were serous carcinomas, 1 was clear cell, and 3 were mixed). Among all the patients, 29 (40.3%) had uterine leiomyomas, and 43 (59.7%) did not.

**Figure 1:**
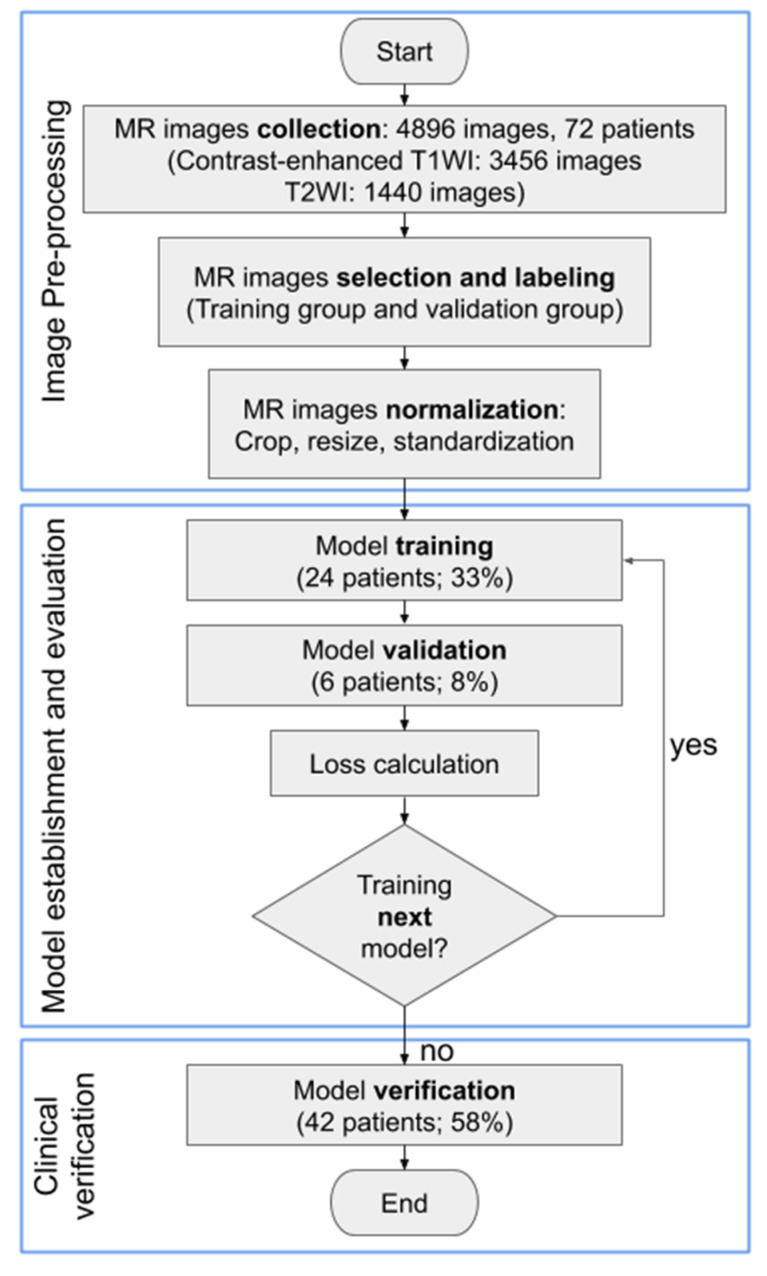
Workflow diagram demonstrating the process and preparation of the MR image dataset and training convolutional neural network models. The consecutive steps of MR image analysis include image upload, convolutional neural network model selection, and diagnosis output.

A total of 4896 MRI slices (3456 slices of contrast-enhanced T1w, and 1440 slices of T2w) with detailed preoperative radiology reports were collected from these 72 patients. Patients were divided into training, validation, and testing groups. The training group was comprised of patients whose results from the radiologists’ diagnoses and from the pathology reports were compatible. One third of the patients (24 patients) were selected as the training group that was used to train the DL model and generate the model parameters. Then, the performance of the model was checked by evaluating the error function using an independent validation group (6 patients). The model that generated the smallest error was selected as the final model. Finally, the test group was comprised of a dataset that was independent of the training group (42 patients plus the 6 patients in the validation group), and this group was used to appraise the accuracy rate of the novel AI-based system (see Figure 1). Two gynecologic oncologists with 25 and 14 years of clinical experience, respectively, were recruited to label the MRI images of each patient, including the contours of the uterus, lesion of the endometrium, and lining of the endometrium. Our research team then double-checked their work. Contrast-enhanced T1w and T2w images were both labeled to provide the AI model with appropriate information for image segmentation and training. Results of the histopathological report were used as a reference to calculate the accuracy rates.

### 2.2. Artificial Intelligence Systems Selection

CNN is an efficient recognition algorithm that is frequently used in image processing and pattern recognition [21,22]. In this study, we used a deep neural network architecture known as U-Net as a model for segmentation of MR images, which consist of equal amount of up- and down-sampling layers. U-Net combines them with the so-called skip connections between opposing convolution and deconvolution layers. It concatenates a contracting path and expansive path while a large number of feature channels during up-sampling allows the propagation of the context information to higher resolution layers [17,23]. The U-Net architecture has proven to be useful for biomedical segmentation applications and medical image analyses [24,25,26,27]. Furthermore, using different methods of weights initialization within the same architecture (known as fine tuning) to initialize the weights for an encoder of the network, VGG11, VGG16, and ResNet34 pre-trained encoder models which converge considerably faster to a steady value and reduce training time in comparison to the non-pre-trained network, were used [28,29]. Based on the findings from previous research [30], the training settings of the aforementioned three architectures included hyperparameters such as batch size, epoch, learning rate, and optimizer that can be adjusted to enhance recognition accuracy. The training results of the AI models were evaluated using data from the validation group [31].

### 2.3. Images Processing and Analysis

In this study, MR images were obtained using 1.5T (Optima MR450W, GE Healthcare, Chicago, IL, USA) and 3T superconducting units (Discovery MR 750, GE Healthcare, Chicago, IL, USA). The imaging protocol for MR imaging scanners typically include sagittal, contrast-enhanced T1w (TR/TE, 501/Minimum ms; section thickness, 4 mm; gap, 1.2 mm; matrix, 288 × 192; and FOV, 280 mm), and sagittal T2w (TR/TE, 5000/90 ms; section thickness, 5 mm; gap, 1 mm; echo-train length, 19; matrix, 320 × 224; and FOV, 240 mm). Since the MR images have different formats and resolutions, initial quality control is important to filter out images with improper formats and low resolutions. We cropped all the raw images into 896 × 896 pixel resolution by using the equation for altered resolution: *P′i* = (*Pi* − *P*mean)/*P*std. Here, *Pi* represents each pixel, *P*mean and *P*std are the mean and standard deviation of all pixels, respectively, and *P′i* is the resulting altered pixel. Moreover, to improve DL efficiency, data augmentation was conducted by multiplying, horizontally flipping, vertically flipping, and using affine transformation on all MR images. Thus, multiple images were derived from the original ones used for AI model training. The augmented dataset was used only for training and not for validation or testing [32]. Thereafter, segmentation was carried out, which involves assigning a label to every pixel in an image such that pixels with the same label share certain characteristics [33]. After processing the MR images, the training and validation groups were used to establish and validate the models, respectively. We used Intersection over Union (IoU) to evaluate the performance of the AI model. IoU is an evaluation metric used to measure the accuracy of an object detector on a particular dataset, which is often used for evaluation the performance of CNN detectors.

### 2.4. Establishing AI Models

**Figure 2:**
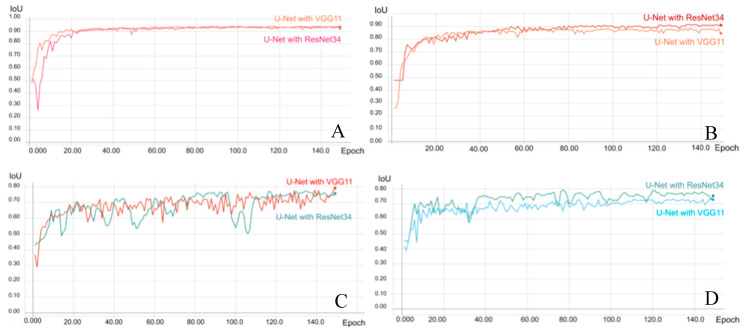
Performance and validation curves for each architecture of the trained convolutional neural network (CNN) models (VGG11 and Resnet34) on MRI. The prediction (intersection over union) score of CNN models in reading the uterus on contrast-enhanced T1w (**A**) and T2w (**B**). The prediction score of CNN models in reading endometrium on contrast-enhanced T1w (**C**) and T2w (**D**).

**Figure A1:**
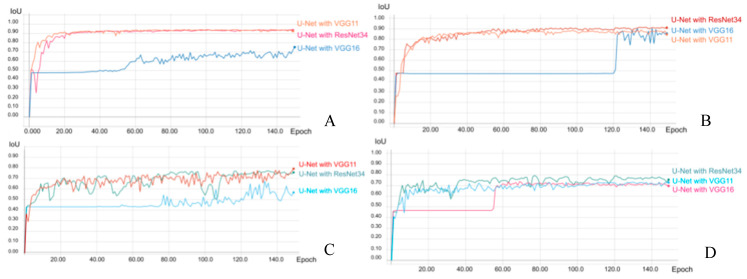
Performance and validation curves for all three architectures of the trained CNN models (VGG11, VGG16 and Resnet34) on MRI. The prediction (IoU) score of CNN models in reading the uterus on contrast-enhanced T1w (**A**) and T2w (**B**). The prediction (IoU) of CNN models in reading endometrium on contrast-enhanced T1w (**C**) and T2w (**D**).

After testing several different models, the U-Net with ResNet34, VGG16, and VGG11 encoders, pre-trained on ImageNet architectures, was used to establish the CNN-based AI models. The above models were established using a QNAP TS-2888X Linux based server with an Intel Xeon CPU, four GPU cards, and 512 GB available RAM for training and validation. During the training process, the original MR and mask images were initially adjusted to have the same size and resolution as the training input images, and the MR images of endometrium and uterus were learned through AI training. The layers were trained by a stochastic gradient descent in a relatively small batch size (16 images) because of the variations in the size and shape of the uterus and endometrial lesions, with a learning rate of 0.001. To determine the best model, the training for all categories was performed for 150 epochs and the loss was calculated using the Dice-coefficient loss function, rather than by the cross entropy loss function, because of its advantage in solving the problem of disparity between the size of the endometrium and non-endometrial areas in the image (Figure A1). After adjusting the parameters, segmentation of the uterus and endometrial lesions in contrast-enhanced T1w exhibited significant and superior performance for the U-Net with VGG11 model and achieved 94.20% and 79.16% of the mean IoU, respectively (Table A1, Table A4 and Table A5). However, the U-Net with ResNet34 model for segmentation training of T2w exhibited better performance than the other models and achieved 91.66% and 79.31% of the mean IoU of the uterus and endometrial lesion, respectively (Table A2, Table A6 and Table A7). The parameters of the best model were selected (see Figure 2) and used for the validation, and these are listed in Table A3.

### 2.5. Statistical Analysis

To determine the differences between diagnoses by the radiologists and AI interpretations, Chi-square tests were used to identify whether two categorical variables were independent of each other, including the “accuracy,” “over-staged/under-staged,” and “whether the concomitant conditions (coexisting uterine leiomyoma, different histology types) affect the diagnoses.” For continuous variables, Pearson correlation was applied to examine the positive or negative relationships between the degree (depth) of myometrial invasion from the pathology reports and the degree (depth) of myometrial invasion interpreted by AI. These two variables were both measured as fractions (myometrial invasion over myometrial thickness). Box and whisker plots were used to compare the accuracy of diagnoses made by radiologists and those made by AI (the center, spread, and overall range of the depth of myometrial invasion were used as determinants), and a one-way analysis of variance (ANOVA) was used to generate the *F*-statistics and *p*-value (α = 0.05 was the standard used to determine any significant differences). This study used STATA 14 software (StataCorp Limited Liability Company, College Station, TX, USA) for statistical analyses.

### 2.6. Ethical Approval

Our research was a retrospective study using the MRI images, pathological reports, and other demographic information of patients. All data/samples were fully anonymized before we accessed them. Only serial numbers were associated with the collected data/samples. We could not identify any individual based on the serial numbers. The data collection period was between January 2019 to November 2019. The IRB approved our study as “Low Risk” (IRB No.: 1-107-05-165).

## 3. Results

### 3.1. Verification of the Final Model

**Figure 3:**
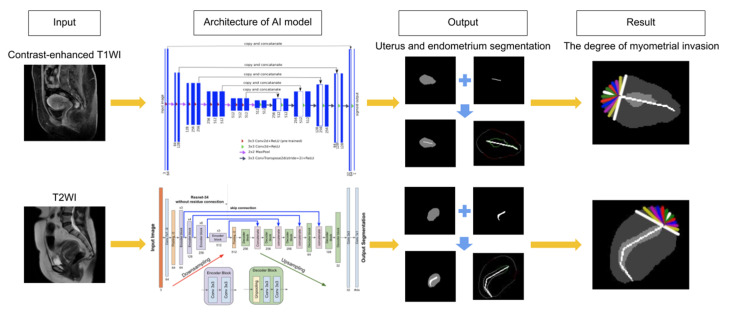
Consecutive steps of MR image analysis include image upload, interpretation of image by AI, and diagnosis output. The architecture of our AI model includes convolutional neural networks consisting of convolution layers, max pooling layers, and a fully connected layer. Each layer extracts different image features; subsequently, all of the extracted features are integrated. The result includes the depth of myometrial invasion and stage classification (FIGO stage IA or IB).

To verify the established AI model, a total of 48 patients were used, and the AI calculated the depth of myometrial invasion by endometrial cancer and then classified each as stage IA or IB. The architecture of our AI model was adopted from Iglovikov and Shvets [24] and modified from Shvets, Iglovikov, Rakhlin, and Kalinin [25] (see Figure 3). The results were compared with the surgico-pathological findings. The accuracy rates for the contrast-enhanced T1w, T2w, and radiologists were 79.2%, 70.8%, and 77.8%, respectively. The chi-square tests showed that there were no significant differences between the AI interpretations for both T1w and T2w and radiologist’s diagnoses (*p* = 0.856 and *p* = 0.392, respectively) (see Table 2). However, we did notice a relatively higher “over-diagnosis rate” from the radiologists. Among the incompatible cases in T1w, 7 out of 10 were over diagnosed (over-diagnosis rate: 70.0%). Among the incompatible cases in T2w, 9 out of 14 were over diagnosed (over-diagnosis rate: 64.3%). However, for the incompatible cases in radiologists’ diagnoses, 14 out of 16 were over diagnosed (over-diagnosis rate: 87.5%).

### 3.2. Effects of Concomitant Conditions on MR Image Interpretation

In addition to the aforementioned findings, we found that the MR images interpreted by AI were more likely to be inaccurate when the patients had coexisting uterine leiomyoma, (*p* = 0.027 for contrast-enhanced T1w and *p* = 0.12 for T2w). In contrast, coexisting leiomyoma usually did not affect the radiologist’s MRI interpretations (*p* = 0.140). Other than uterine leiomyoma, different histological subtypes did not affect the accuracy of the radiologists (*p* = 0.413) or the AI (*p* = 0.549 for contrast-enhanced T1w; *p* = 0.727 for T2w) (see Table 3).

**Figure 4:**
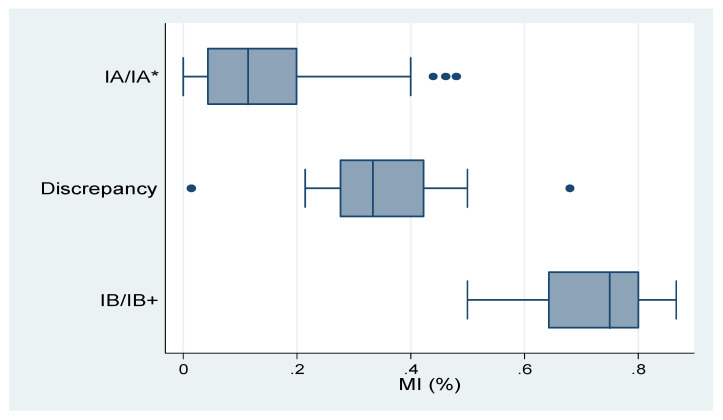
Box and Whisker Plot of data from radiologists′ diagnoses and pathological stages.

**Figure 5:**
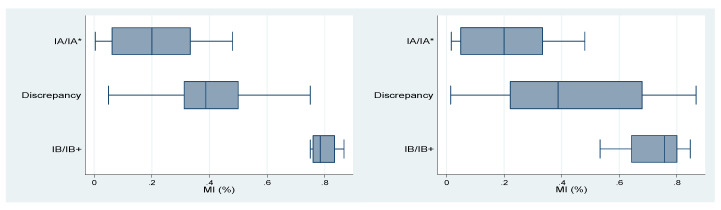
Box and Whisker Plot of data from AI interpretation and pathological stages (contrast-enhanced T1w and T2w).

**Figure A2:**
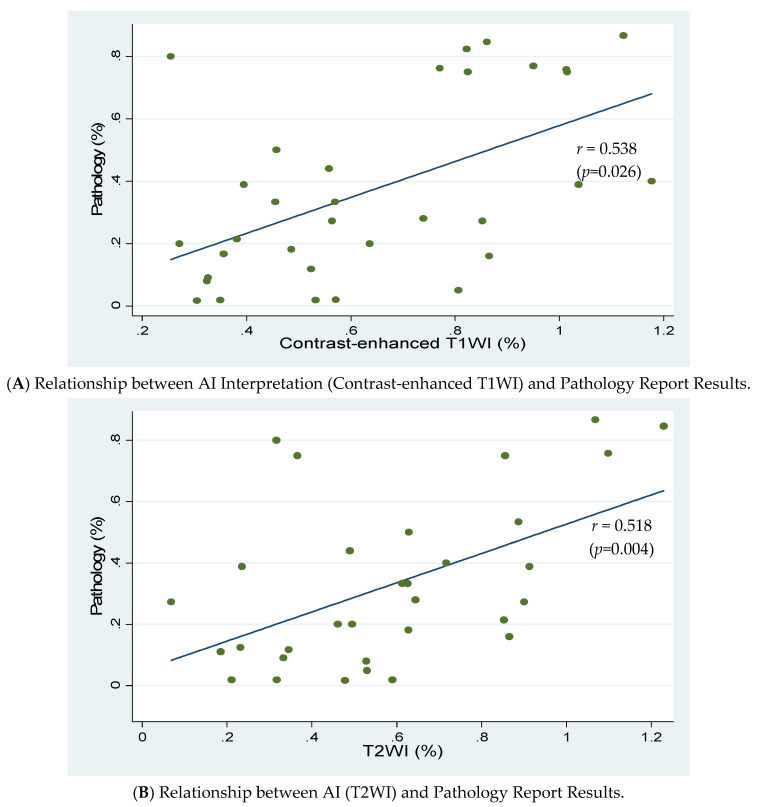
Graphs showing the relationship between pathologic and imaging findings regarding myometrial invasion. The correlation coefficient for contrast-enhanced T1w (**A**) and T2w (**B**) of a patient without uterine leiomyoma is 0.538 and 0.518, respectively.

In addition, we found a positive correlation between the depth of myometrial invasion (the percentage of myometrial invasion over myometrial thickness) from the pathology report and that interpreted by AI (*r* = 0.54 for contrast-enhanced T1w, *p* = 0.026; *r* = 0.52 for T2w, *p* = 0.004) (see Figure A2). We also found that the distribution of the percentage of myometrial invasion may lead to discrepancies between the stages diagnosed by radiologists or AI. Results are presented in Table 4 and Table 5. Table 4 and Figure 4 show the results of radiologists’ diagnoses. As shown in the table, when the degree of myometrial invasion was relatively small or large, radiologists were less likely to make incorrect decisions. However, when the depth of myometrial invasion was around 50%, radiologists’ diagnoses tended to be incompatible with the pathology reports. The box and whisker plots display the distribution of myometrial invasion. Table 5 and Figure 5 show the results of AI interpretation. Similarly, when the degree of myometrial invasion was at the two extremes, AI was more likely to generate a correct answer; however, when the depth of myometrial invasion was in the middle range (50%), AI also generated incompatible results. Particularly, the range for AI to make discrepant results for the T2w (from 1.5% to 86.7%) was significantly wider than that for the T1w or for the radiologists. Results of the ANOVA showed that the closer the depth of myometrial invasion was to 50%, the easier it was for both radiologists (*F*-value = 99.06, *p* < 0.001) and AI (*F*-value = 44.46, *p* < 0.001 for contrast-enhanced T1w; *F*-value = 17.68, *p* < 0.001 for T2w) to provide incorrect diagnoses. We found the results reasonable since the ability to determine the pre-operative MRI stages based, mainly, on personal expertise and experience which vary from person to person. On the other hand, using AI to determine the depth of myometrial invasion may also be affected by various pathological factors, such as irregular endomyometrial junction inside single uterus, hematometra, endometrial polyps, exophytic tumor growth, adenomyosis or extensive leiomyomas. These factors may inevitably cause both human beings and AI to come up with incorrect diagnoses, especially when there is a clear cut-off value, below or above 50% myometrial invasion.

## 4. Discussion

We compared the accuracy rates of the radiologists’ diagnoses and AI interpretations based on the depth of myometrial invasion. The results indicated that the AI interpretations for both contrast-enhanced T1w and T2w were similar to radiologists’ diagnoses. Although small differences exist, they were not statistically significant. However, we found that the closer the depth of myometrial invasion was to 50%, the easier it was for both the radiologists and AI to provide incorrect judgements. However, compared with AI, radiologists were more likely to “over-stage” the results from IA to IB. We believe these findings shed light on the fact that human beings tend to act conservatively when making critical decisions. In clinical practice, when facing with a situation where it is necessary to choose the lesser of two evils, radiologists would rather let the patients receive more evaluations or treatments than receive insufficient ones. More treatments usually include more extensive surgeries (lymph nodes dissection), radiation therapy, and/or chemotherapy. However, receiving more treatments are not always beneficial, and they also come with additional risks. Patients are more likely to suffer from surgical complications or therapy-related complications.

In addition, we found that when patients had coexisting leiomyoma, AI was more likely to provide incorrect interpretations. However, the coexisting leiomyoma did not affect the radiologists’ judgements. We believe that was because the myometrial compression from a leiomyoma or bulky polypoid tumor would lead to unclear boundaries between the tumor and myometrium, which would make it difficult for the AI to calculate the depth. The histological types of endometrial carcinoma, on the contrary, did not affect the radiologists or AI. Such findings suggested that the primary role of MRI used in gynecologic oncology is in delineating the extent of the disease, not for analyzing the morphological features or histological types [34].

Still, AI technology is potentially useful especially in hospitals without radiologists specializing in gynecology. There are several benefits of using AI to predict myometrial invasion before surgery. First, it can affect the choice of surgical approach methods, and second, it can be used to determine if lymphadenectomy is necessary. In the early stages, patients have the chance to choose either exploratory laparotomy or micro-invasive laparoscopic surgery. The result of a Gynecologic Oncology Group LAP2 trial for early stage endometrial cancer reported favorable recurrence and survival outcomes of laparoscopy surgical staging [35]. In another Cochrane review published in 2018 [36], the key results revealed no difference in perioperative mortality risk, overall survival, and disease-free survival between laparoscopy and laparotomy. Furthermore, laparoscopy is associated with significantly shorter hospital stays. In hospitals without radiologists specializing in gynecology, AI technology could help identify low-risk patients with stage IA disease, and therefore, the gynecological oncologist may feel more comfortable performing laparoscopic surgery.

In addition, the necessity of routine lymphadenectomy in staging surgery has been widely debated. The Mayo group described the criteria of patients with a low risk of nodal disease spread and a high disease-free survival rate: grade 1 to 2 tumors, less than 50% myometrial invasion, and tumor size less than 2 cm [37]. In GOG Lap 2 trial, in 971 patients with type 1 endometrioid carcinoma, 40% met Mayo low-risk criteria and only 0.8% (3/389) had positive nodes. Therefore, with careful selection of low risk patients, lymphadenectomy may be safely avoided, which reduces surgery-related morbidity such as lower limb lymphedema or intra-abdominal lymphocele formation. In hospitals without radiologists specializing in gynecology and gynecologists specializing in oncologic surgery, with help of AI pre-operative diagnosis, a general gynecological surgeon could perform simple hysterectomy, bilateral salpingo-oophorectomy without lymphadenectomy for selective low-risk patients. In remote area with limited medical resources, AI pre-operative diagnosis will reduce the need of transferring low-risk patients to tertiary medical center.

We noted a few potential limitations of our study. First, our datasets were built based on cases from one institution. The diagnoses made using MRI and pathology were based on personal expertise and experience, which exhibit individual differences. Although the Tri-Service General Hospital is a medical center in Taipei, we still cannot eliminate the possibility of that there is bias in the data. Second, the key imaging sequence used to assess uterine cavity tumors involves choosing a sagittal T2w. However, a multiparametric approach which combines T2w and contrast-enhanced T1w along with different planes may represent the most comprehensive approach to assess tumor spread. Also, our study did not analyze the diffusion-weighted imaging or the MR spectroscopy, which may could serve as one potential research subject in diagnosing endometrial cancer. Training AI from a two-dimensional approach (largest cross-sectional tumor area) to a three-dimensional approach (whole tumor) could lead to differences in establishing the AI-based model or affect the accuracy of the interpretation of the results, especially for results obtained by assessing only the sagittal plane of T2w and contrast-enhanced T1w [38,39]. Third, different CNN architectures might be suitable for the interpretation of different diseases’ images, and thereby selecting and establishing appropriate architectures might provide slightly different results [23,24,25,26]. Fourth, the impact of ethnic differences was not examined because all the patients in this study were Taiwanese. Therefore, using AI to determine the depth of myometrial invasion still has its weak spot given the limited cases using for AI training regarding the endometrial cancer. The AI’s decision cannot be final at this point. However, if more diverse cases can be used for deep learning in the future, or if we can develop a multicenter database for this purpose, we may further enhance the validity of the AI and improve the quality of our health care. Alternatively, we could further design a prospective randomized study to identify a population of patients with endometrial cancer to examine the efficacy of AI-assisted method. Our paper, as a pilot study in this uncharted territory, shows that using AI as an assist to interpret the depth of myometrial invasion of MRI is indeed advantageous to prevent the high interobserver variability among radiologists [18].

## 5. Conclusions

In summary, although current AI technology may not be able to replace the expertise and experience of physicians, AI could be used as an auxiliary resource. From the perspective of balancing human proactive errors and passive errors, it could be beneficial for the physicians to have a “second opinion” from the AI technology before making critical judgement calls on endometrial cancer. Our research is the first attempt to use AI technology to evaluate the invasion depth of myometrium in early stage endometrial cancers. There have not been any publications about AI applications in endometrial cancers. In the future, refinement of selection and establishment of a deep learning model with a larger image database are essential to improve the accuracy. We believe artificial intelligence has the potential to assist radiologists or serve as a reasonable alternative for pre-operative evaluation of the myometrial invasion depth of stage I endometrial cancers.

## Figures and Tables

**Table 1:** Clinical and pathologic characteristics of all patients.

Characteristics	*n* = 72
Age (year) [mean ± SD ^a^] (range)	59.7 ± 9.08 (39–85)
Menopausal status	
Postmenopausal	63 (87.5%)
Premenopausal	9 (12.5%)
ECOG ^b^ performance status	
0	54
1	18
2	0
3	0
4	0
FIGO ^c^ Stage	
IA	53 (73.6%)
IB	19 (26.4%)
Histology subtype	
Type I	
Grade 1 endometrioid	27 (37.5%)
Grade 2 endometrioid	32 (44.4%)
Type II	
Grade 3 endometrioid	4 (5.6%)
Serous	5 (6.9%)
Clear cell	1 (1.4%)
Mixed	3 (4.2%)
Histology grade	
1	27 (37.5%)
2	32 (44.4%)
3	13 (18.1%)
Uterine leiomyomas	
Present	29 (40.3%)
Absent	43 (59.7%)

Characteristics are presented based on the pathology reports. ^a^ SD: standard deviation. ^b^ ECOG: Eastern Cooperative Oncology Group. ^c^ FIGO: International Federation of Gynecology and Obstetrics.

**Table 2:** Accuracy rates of AI and radiologists.

Results	Pathology Report		Accuracy Rates
	IA	IB	
AI Interpretation			
Contrast-enhanced T1w			79.2% (38/48)
<50% Invasion	30 (compatible)	3 (under diagnosed)	
≥50% Invasion	7 (over diagnosed)	8 (compatible)	
T2w			70.8% (34/48)
<50% Invasion	29 (compatible)	5 (under diagnosed)	
≥50% Invasion	9 (over diagnosed)	5 (compatible)	
Radiologists’ Diagnoses			77.8% (56/72)
IA	39 (compatible)	2 (under diagnosed)	
IB	14 (over diagnosed)	17 (compatible)	

Chi-square test results: For Contrast-enhanced T1w and Radiologists: ***χ*^2^** = 0.033, *p* = 0.856; For T2w and Radiologists: ***χ*^2^** = 0.738, *p* = 0.392.

**Table 3:** Influence of concomitant conditions on the accuracy rates of AI and radiologists.

Pathology Report						
Results	IA	IB	IA	IB	Accuracy Rates	*p*-Value
Uterine leiomyoma	+		−		+/−	
AI Interpretation						
Contrast-enhanced T1w					60%/87.9%	0.027
<50% Invasion	9 *	1	21 *	2		
≥50% Invasion	5	0 *	2	8 *		
T2w					56.3%/78.1%	0.115
<50% Invasion	8 *	1	21 *	4		
≥50% Invasion	6	1 *	3	4 *		
Radiologists’ Diagnoses (MR stage)					69%/83.7%	0.140
IA	16 **	1	23 **	1		
IB	8	4 **	6	13**		
Histology	Type I		Type II		Type I/II	
AI Interpretation						
Contrast-enhanced T1w					81.1%/72.7%	0.549
<50% Invasion	26 *	2	4 *	1		
≥50% Invasion	5	4 *	2	4 *		
T2w					71.1%/70%	0.727
<50% Invasion	25 *	4	4 *	1		
≥50% Invasion	7	2 *	2	3 *		
Radiologists’ Diagnoses (MR stage)					79.7%/69.2%	0.413
IA	35 **	1	4 **	1		
IB	11	12 **	3	5 **		

* “compatible” between pathology report and AI interpretation; ** “compatible” between pathology report and radiologists’ diagnoses.

**Table 4:** Results of radiologists′ diagnoses and pathological stages.

Results	Min	Q1	Median	Q3	Max
IA/IA * (compatible)	0	0.043	0.114	0.2	0.48
Discrepancy	0.015	0.276	0.333	0.422	0.68
IB/IB+ (compatible)	0.5	0.643	0.75	0.8	0.867

IA/IA *: pathological stage/clinical stage; IB/IB+: pathological stage/clinical stage; MI: myometrial invasion; ANOVA: *F*-value = 99.06, *p* = 0.000.

**Table 5:** Results of AI interpretation and pathological stages (contrast-enhanced T1w and T2w).

Contrast-Enhanced T1w						T2w					
	Min	Q1	Median	Q3	Max		Min	Q1	Median	Q3	Max
IA/IA * (Compatible)	0.004	0.063	0.2	0.333	0.48	IA/IA * (compatible)	0.017	0.05	0.2	0.333	0.48
Discrepancy	0.05	0.313	0.388	0.5	0.75	Discrepancy	0.015	0.222	0.388	0.68	0.867
IB/IB+ (Compatible)	0.75	0.76	0.785	0.835	0.867	IB/IB+ (compatible)	0.533	0.643	0.758	0.8	0.846

IA/IA *: pathological stage/clinical stage; B/IB+: pathological stage/clinical stage; MI: myometrial invasion; ANOVA: Contrast-enhanced T1w: *F*-value = 44.46, *p* = 0.000; T2w: *F*-value = 17.68, *p* = 0.000.

## Appendix A

**Table A1:** Verification summary of the performance of three AI models on contrast-enhanced T1w using the validation dataset. The parameters of accuracy, sensitivity and specificity for both uterus and endometrium are shown.

	U-Net with VGG 11	U-Net with VGG 16	U-Net with ResNet34
Accuracy (Uterus)	96.83%	76.83%	97.06%
Accuracy (Endometrium)	85.96%	73.13%	84.31%
Mean loU (Uterus)	94.20%	75.30%	93.92%
Mean loU (Endometrium)	79.16%	67.54%	77.23%
Mean Dice (Uterus)	96.94%	84.01%	96.78%
Mean Dice (Endometrium)	87.62%	78.22%	86.24%
Mean Precision (Uterus)	97.05%	97.44%	96.51%
Mean Precision (Endometrium)	89.54%	88.86%	88.53%
Mean Recall (Uterus)	96.83%	76.83%	97.06%
Mean Recall (Endometrium)	85.96%	73.13%	84.31%
Mean Specificity (Uterus)	96.83%	76.83%	97.06%
Mean Specificity (Endometrium)	85.96%	73.13%	84.31%

**Table A2:** Verification summary of the performance of three AI models on T2w using the validation dataset. The parameters of accuracy, sensitivity and specificity for both uterus and endometrium are shown.

	U-Net with VGG 11	U-Net with VGG 16	U-Net with ResNet34
Accuracy (Uterus)	95.80%	95.49%	96.86%
Accuracy (Endometrium)	83.60%	82.88%	87.34%
Mean loU (Uterus)	88.78%	90.61%	91.66%
Mean loU (Endometrium)	73.18%	73.63%	79.31%
Mean Dice (Uterus)	93.75%	94.88%	95.49%
Mean Dice (Endometrium)	82.60%	82.95%	87.56%
Mean Precision (Uterus)	91.90%	94.28%	94.20%
Mean Precision (Endometrium)	81.67%	83.02%	87.78%
Mean Recall (Uterus)	95.80%	95.50%	96.86%
Mean Recall (Endometrium)	83.60%	82.88%	87.34%
Mean Specificity (Uterus)	95.80%	95.49%	96.86%
Mean Specificity (Endometrium)	83.60%	82.88%	87.34%

**Table A3:** Performance of each architecture of the trained CNN models on contrast-enhanced T1w and T2w. The parameters of learning rate, batch size, total epochs, epochs with the best value of IoU and mean IoU of both the uterus and endometrium are shown.

	Contrast-Enhanced T1w	T2w
Type	Uterus / Endometrium	
Architecture	U-Net with VGG 11	U-Net with ResNet34
Optimizer	Adam	
Learning Rate	1e-4	
Batch size	16	
Total number of epochs run during training	150	
Epochs with the maximum value of loU (best model)	89/95	137/77
Mean loU of validation of Uterus/Endometrium (best model)	92.64%/80.40%	91.66%/79.31%

**Table A4:** Parameters and performance of all three architectures on “contrast-enhanced T1w” of the “uterus.”

Loss Weight	Batch Size	Loss	Learning Rate	Architecture (U-Net with #)	Data	Augmentation	Best Epoch	Mean IoU (%)	IoU 0 (%)	IoU 1 (%)	Mean Dice (%)	Dice 0 (%)	Dice 1 (%)
10	16	dice	0.0001	VGG11	T1WI	TRUE	101	94.20	99.45	88.95	96.94	99.73	94.15
10	16	Cross Entropy	0.0001	VGG11	T1WI	TRUE	98	94.10	99.44	88.76	96.88	99.72	94.04
10	16	dice	0.0001	VGG16	T1WI	TRUE	150	75.30	97.70	52.89	84.01	98.83	69.19
10	16	dice	0.0001	ResNet34	T1WI	TRUE	131	93.92	99.42	88.42	96.78	99.71	93.86
10	16	dice	0.0001	ResNet34	T1WI	FALSE	133	92.06	99.24	84.87	95.72	99.62	91.82
10	32	dice	0.0001	ResNet34	T1WI	FALSE	115	91.62	99.18	84.06	95.46	99.59	91.34
10	32	dice	0.0001	ResNet34	T1WI	TRUE	140	93.66	99.40	87.93	96.64	99.70	93.57
10	32	Cross Entropy	0.0001	ResNet34	T1WI	TRUE	104	91.17	99.26	83.08	95.19	99.63	90.76

**Table A5:** Parameters and performance of all three architectures on “contrast-enhanced T1w” of the “endometrium.”.

Loss Weight	Batch Size	Loss	Learning Rate	Architecture (U-Net with #)	Data	Augmentation	Best Epoch	Mean IoU (%)	IoU 0 (%)	IoU 1 (%)	Mean Dice (%)	Dice 0 (%)	Dice 1 (%)
10	16	dice	0.0001	VGG11	T1WI	TRUE	149	79.16	93.74	64.59	87.62	96.77	78.48
10	16	dice	0.0001	VGG11	T1WI	FALSE	32	73.53	91.86	55.19	83.44	95.75	71.13
10	16	dice	0.0001	VGG16	T1WI	TRUE	133	67.54	91.07	44.01	78.22	95.33	61.12
10	16	dice	0.0001	VGG16	T1WI	FALSE	4	47.37	94.73	0.00	48.65	97.29	0.00
10	16	dice	0.0001	ResNet34	T1WI	FALSE	113	75.38	92.34	58.42	84.89	96.02	73.75
10	16	dice	0.0001	ResNet34	T1WI	TRUE	72	79.59	93.82	65.36	87.93	96.81	79.05
10	32	dice	0.0001	ResNet34	T1WI	TRUE	135	77.23	93.13	61.33	86.24	96.44	76.03
10	32	dice	0.0001	ResNet34	T1WI	FALSE	85	75.93	97.12	54.75	84.65	98.54	70.76

**Table A6:** Parameters and performance of all three architectures on “T2w” of the “uterus.”

Loss Weight	Batch Size	Loss	Learning Rate	Architecture (U-Net with #)	Data	Augmentation	Best Epoch	Mean IoU (%)	IoU 0 (%)	IoU 1 (%)	Mean Dice (%)	Dice 0 (%)	Dice 1 (%)
10	16	dice	0.0001	VGG11	T2WI	TRUE	135	88.78	98.92	78.64	93.75	99.46	88.04
10	16	dice	0.0001	VGG11	T2WI	FALSE	59	85.59	98.55	72.64	91.71	99.27	84.15
10	16	dice	0.0001	VGG16	T2WI	TRUE	141	90.61	98.92	82.30	94.88	99.46	90.29
10	16	dice	0.0001	VGG16	T2WI	FALSE	115	87.56	98.70	76.41	92.99	99.34	86.63
10	16	dice	0.0001	ResNet34	T2WI	FALSE	148	85.73	98.52	72.94	91.80	99.25	84.35
10	16	dice	0.0001	ResNet34	T2WI	TRUE	137	91.66	99.19	84.14	95.49	99.59	91.38
10	32	dice	0.0001	ResNet34	T2WI	FALSE	149	78.67	97.53	59.82	86.80	98.75	74.86
10	32	dice	0.0001	ResNet34	T2WI	TRUE	124	89.25	98.92	79.58	94.04	99.46	88.63
5	64	dice	0.00005	ResNet34	T2WI	TRUE	148	83.45	98.16	68.73	90.27	99.07	81.46
5	64	dice	0.0001	ResNet34	T2WI	TRUE	143	88.84	98.88	78.80	93.79	99.43	88.14
5	64	dice	0.0002	ResNet34	T2WI	TRUE	136	90.90	99.12	82.68	95.04	99.56	90.52
10	64	dice	0.00005	ResNet34	T2WI	TRUE	148	82.10	98.00	66.21	89.33	98.99	79.67
10	64	dice	0.0001	ResNet34	T2WI	TRUE	141	87.93	98.77	77.10	93.22	99.38	87.07
10	64	dice	0.0002	ResNet34	T2WI	TRUE	127	90.30	99.02	81.58	94.68	99.51	89.85
20	64	dice	0.00005	ResNet34	T2WI	TRUE	133	86.37	98.62	74.13	92.22	99.31	85.14
20	64	dice	0.0001	ResNet34	T2WI	TRUE	132	89.42	98.96	79.87	94.14	99.48	88.81
20	64	dice	0.0002	ResNet34	T2WI	TRUE	135	91.33	99.16	83.49	95.29	99.58	91.00
10	16	Cross Entropy	0.0001	ResNet34	T2WI	TRUE	134	91.50	99.19	83.80	95.39	99.59	91.19
10	72	Cross Entropy	0.0001	ResNet34	T2WI	TRUE	124	88.57	98.85	78.29	93.62	99.42	87.82
10	72	Cross Entropy	0.0002	ResNet34	T2WI	TRUE	131	90.22	99.03	81.40	94.63	99.51	89.74
10	72	Cross Entropy	0.0004	ResNet34	T2WI	TRUE	80	90.07	99.04	81.11	94.54	99.52	89.57

**Table A7:** Parameters and performance of all three architectures on “T2w” of the “endometrium.”

Loss Weight	Batch Size	Loss	Learning Rate	Architecture (U-Net with #)	Data	Augmentation	Best Epoch	Mean IoU (%)	IoU 0 (%)	IoU 1 (%)	Mean Dice (%)	Dice 0 (%)	Dice 1 (%)
10	16	dice	0.0001	VGG11	T2WI	FALSE	18	77.88	94.63	61.12	86.56	97.24	75.87
10	16	dice	0.0001	VGG11	T2WI	TRUE	43	79.07	95.23	62.90	87.39	97.56	77.23
10	16	dice	0.0001	VGG16	T2WI	FALSE	69	73.63	95.56	51.70	82.95	97.73	68.16
10	16	dice	0.0001	VGG16	T2WI	TRUE	3	46.67	93.33	0.00	48.28	96.55	0.00
10	16	dice	0.0001	ResNet34	T2WI	TRUE	77	79.31	95.39	63.24	87.56	97.64	77.48
10	64	dice	0.00005	ResNet34	T2WI	TRUE	136	77.87	95.17	60.57	86.48	97.52	75.44
20	64	dice	0.00005	ResNet34	T2WI	TRUE	143	78.31	95.35	61.27	86.80	97.62	75.98
